# The Hypnotic, Anxiolytic, and Antinociceptive Profile of a Novel µ-Opioid Agonist

**DOI:** 10.3390/molecules22050800

**Published:** 2017-05-16

**Authors:** Guilherme Carneiro Montes, Bianca Nascimento Monteiro da Silva, Bismarck Rezende, Roberto Takashi Sudo, Vitor Francisco Ferreira, Fernando de Carvalho da Silva, Angelo da Cunha Pinto, Bárbara Vasconcellos da Silva, Gisele Zapata-Sudo

**Affiliations:** 1 Programa de Pesquisa em Desenvolvimento de Fármacos, Instituto de Ciências Biomédicas, Universidade Federal do Rio de Janeiro, Rio de Janeiro RJ 21941-902, Brazil; montes.guilherme@gmail.com (G.C.M.); bismarckrezende@gmail.com (B.R.); rtakashisudo@gmail.com (R.T.S.); 2 Instituto de Química, Universidade Federal do Rio de Janeiro, Rio de Janeiro RJ 21941-909, Brazil; bianca_qnascimento@yahoo.com.br (B.N.M.d.S.); angelocpinto@gmail.com (A.d.C.P.); barbara.iq@gmail.com (B.V.d.S.); 3 Instituto Nacional de Ciência e Tecnologia de Fármacos e Medicamentos (INCT-INOFAR), Rio de Janeiro RJ 21941-971, Brazil; 4 Instituto de Química, Universidade Federal Fluminense, Niterói RJ 24020-150, Brazil; cegvito@vm.uff.br (V.F.F.); gqofernando@vm.uff.br (F.d.C.d.S.)

**Keywords:** novel µ-opioid agonist, hypnosis, antinociception, anti-inflammatory effect, mice

## Abstract

5′-4-Alkyl/aryl-1*H*-1,2,3-triazole derivatives **PILAB 1**–**12** were synthesized and a pharmacological screening of these derivatives was performed to identify a possible effect on the Central Nervous System (CNS) and to explore the associated mechanisms of action. The mice received a peritoneal injection (100 µmol/kg) of each of the 12 PILAB derivatives 10 min prior to the injection of pentobarbital and the mean hypnosis times were recorded. The mean hypnosis time increased for the mice treated with **PILAB 8**, which was prevented when mice were administered CTOP, a µ-opioid antagonist. Locomotor and motor activities were not affected by **PILAB 8**. The anxiolytic effect of **PILAB 8** was evaluated next in an elevated-plus maze apparatus. **PILAB 8** and midazolam increased a percentage of entries and spent time in the open arms of the apparatus compared with the control group. Conversely, a decrease in the percentages of entries and time spent in the closed arms were observed. Pretreatment with naloxone, a non-specific opioid antagonist, prior to administration of **PILAB 8** exhibited a reverted anxiolytic effect. **PILAB 8** exhibited antinociceptive activity in the hot plate test, and reduced reactivity to formalin in the neurogenic and the inflammatory phases. These data suggest that **PILAB 8** can activate µ-opioid receptors to provoke antinociceptive and anti-inflammatory effects in mice.

## 1. Introduction

**Figure 1:**
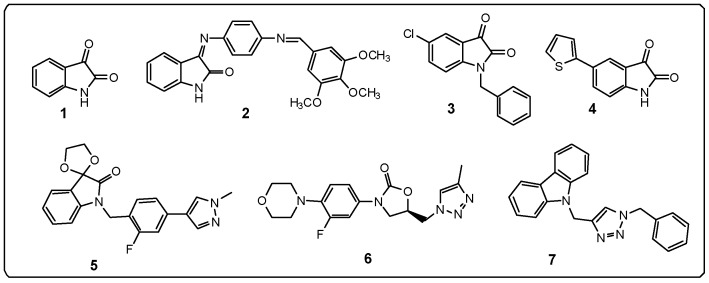
Structure of isatins **1**–**5** and triazoles derivatives **6**–**7**.

Pain is defined as an unpleasant sensory feeling that results from activation of sensory nerve endings in response to a stimulus which can vary among individuals due to emotional state, gender, ethnicity, anxiety level, early experiences and memories [1,2,3,4,5,6]. Pain management to improve quality of life depends on agents with analgesic properties such as non-narcotic analgesics (e.g., acetominophen and aspirin), narcotic analgesics (opioids), and other drug classes, including antidepressants and anticonvulsants [7,8]. However, reduction of pain is limited, which is the main reason for the development of new therapies [9]. Isatin (1*H*-indole-2,3-dione, **1**, Figure 1), is distributed among various regions of the brain and heart, thereby indicating that this substance has important physiological functions. Isatin and its derivatives act by inhibiting the enzyme monoamine oxidase B (MAO-B) in the brain and reducing the formation of cyclic guanosine monophosphate (cGMP). In addition, isatin interacts with benzodiazepine receptors such as the ligand-gated ion channel receptor γ-aminobutyric acid (GABA), which can mediate sedative, hypnotic, analgesic, and other important effects on the central nervous system (CNS) [10,11,12,13]. Several studies have demonstrated that isatin derivatives could promote actions on the CNS. Thus, compound **2** (Figure 1) was described as anticonvulsant agent [14], compounds **3** and **4** (Figure 1) inhibited human GABA transporter 3 [15] and compound **5** (Figure 1) produced a positive allosteric modulation of human muscarinic M1 receptor [16].

Triazoles containing oxazolidinone rings mediate inhibition of MAO and the presence of the methyl group on the triazolic ring (e.g., compound **6**, Figure 1) provides selectivity for the MAO-B isoform [17]. Carbazole derivatives containing the *N-*benzyl-1,2,3-triazole moiety like **7** (Figure 1) also exhibit significant anti-acetylcholinesterase activity (IC_50_ ≤ 3.8 μM). Meanwhile, molecular modeling studies have shown the existence of π-π interactions between the triazole ring and Tyr334 in the anionic binding site of the enzyme [18].

**Figure 2:**
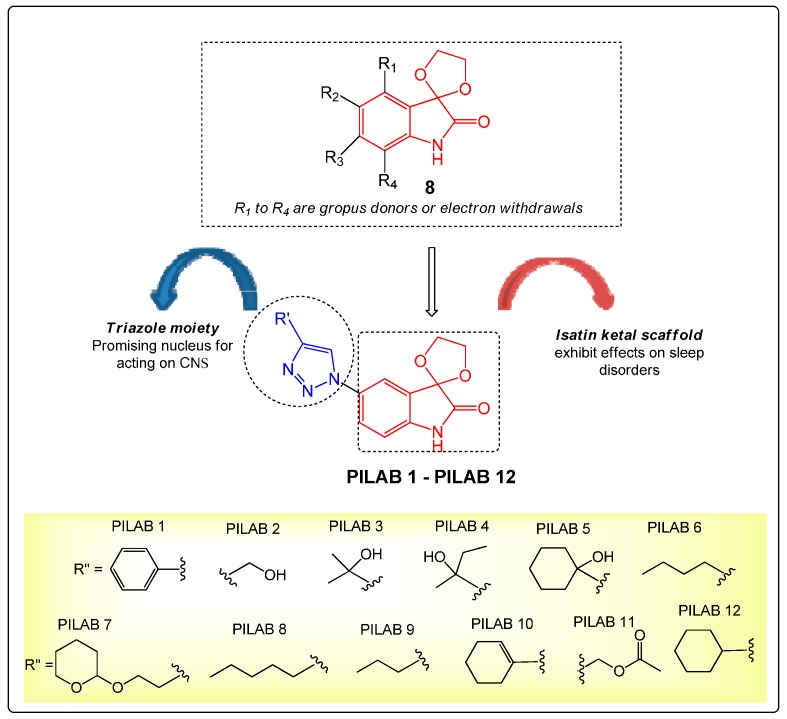
Design of isatin-triazoles **PILAB 1**–**PILAB 12** with potential CNS activity, and structure of dioxolane ketal isatin derivatives **8** previously evaluated by our research group.

Previously, we reported that dioxolane ketal isatin derivatives such as **8** (Figure 2) exhibited beneficial effects on sleep disorders and represented an alternative for the maintenance of anesthesia [19]. Fix figure: R1 and R4 are electron donating or withdrawing groups; there is no R″ in the Figures.

The compounds were initially synthesized using a 1,3-dipolar cycloaddition reaction catalyzed by acetic acid [20] and then, the route was improved using ultrasound irradiation, which reduced the reaction time to 5 min, with no need of purification using column chromatography [21]. Thus, the present work reports the action of isatin-type 5′-4-akyl/aryl-1*H*-1,2,3-triazoles **PILAB 1**–**PILAB 12** on the CNS through the evaluation of their sedative-hypnotic profile. The compound with optimal activity was selected and further evaluated to elucidate the mechanisms involved in its action.

## 2. Results

### 2.1. Effect of PILABs on Pentobarbital-Induced Sleep

**Figure 3:**
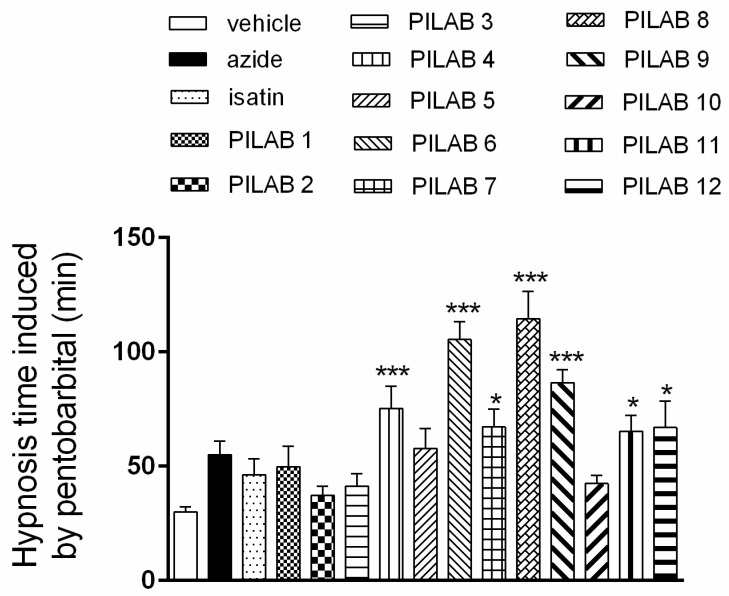
Effect of PILABs (100 μmol/kg) on the duration of pentobarbital-induced sleep. Mice received i.p. injections of the PILABs indicated 30 min prior to an i.v. injection of sodium pentobarbital (20 mg/kg). Hypnosis time was recorded based on the loss and recovery of the righting reflex. Data are expressed as the mean ± SEM (*n* = 10). * *p* < 0.05, and *** *p* < 0.001 compared to the vehicle-treated group, one-way ANOVA followed by Dunnett’s multiple comparison test for parametric measures.

As illustrated in Figure 3, the duration of pentobarbital-induced sleep increased from 30.0 ± 2.2 s in the animals that received vehicle to 75.1 ± 9.9 s, 105.4 ± 7.8 s, 67.2 ± 7.7 s, 114.6 ± 11.8 s, 86.6 ± 5.7 s, 65.3 ± 6.8 s and 66.8 ± 11.4 s when the mice were treated with **PILAB 4**, **6**, **7**, **8**, **9**, **11** and **12**, respectively.

### 2.2. Hypnosis Following i.v. Injections of the Various PILABs and an Evaluation of the Mechanism of Action

**Figure 4:**
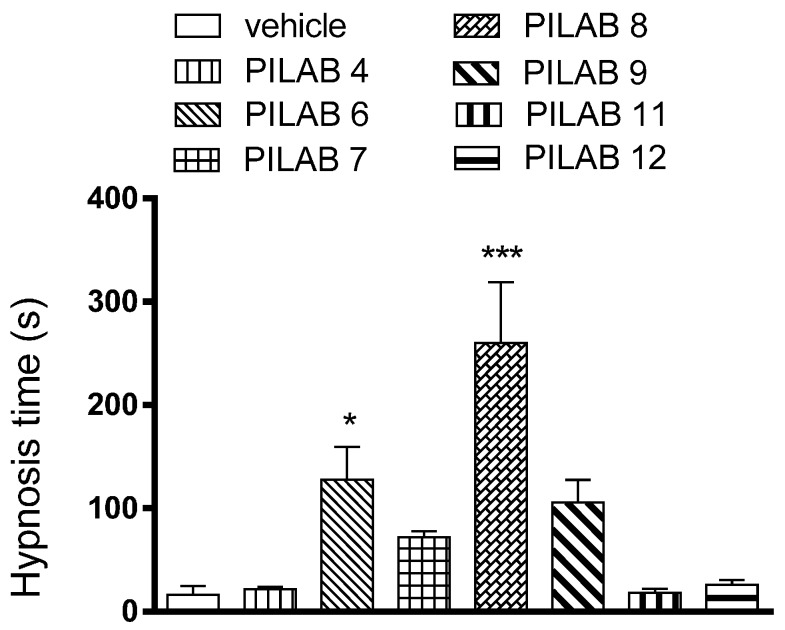
Hypnosis time following i.v. administration of the PILABs (100 μmol/kg). Time intervals between loss and recovery of righting reflex were recorded. Data are expressed as the mean ± SEM (*n* = 10). * *p* < 0.05 and *** *p* < 0.001 compared to the vehicle-treated group, one-way ANOVA followed by Dunnett's multiple comparison test for parametric measures.

**PILAB 4**, **6**, **7**, **8**, **9**, **11** and **12** were selected for testing if they alone could induce hypnosis after intravenous injection. **PILAB 6** and **PILAB 8** enhanced the hypnosis time from 12.2 ± 6.3 s (vehicle) to 127.8 ± 31.7 s and 260.0 ± 58.9 s, respectively (Figure 4).

**Figure 5:**
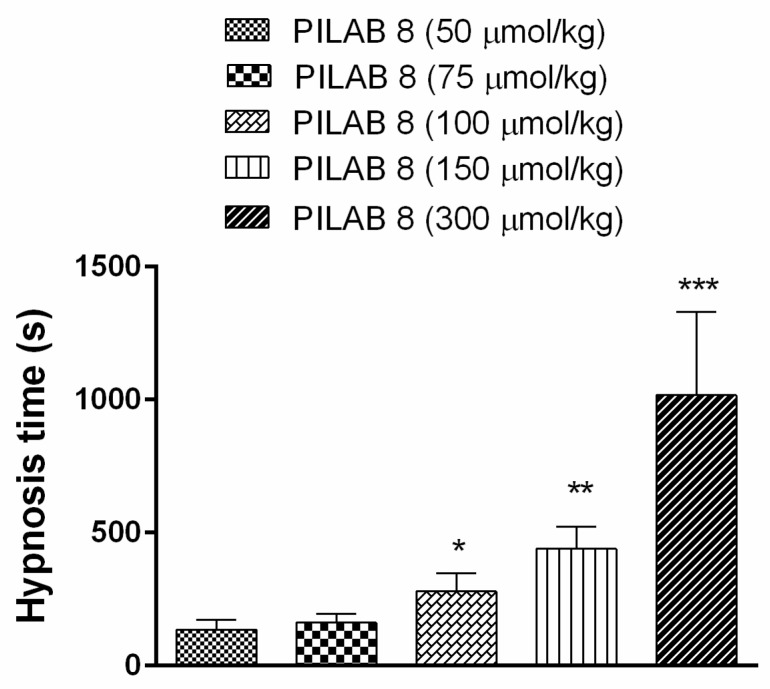
Hypnosis time following the i.v. administration of varying doses of **PILAB 8**. Time intervals between loss and recovery of the righting reflex were recorded. Data are expressed as the mean ± SEM (*n* = 6). * *p* < 0.05 and ** *p* < 0.01 and *** *p* < 0.001 compared to **PILAB 8** (50 μmol/kg), one-way ANOVA followed by Student’s unpaired *t*-test.

**PILAB 8** promoted hypnosis in a dose dependent manner because when it was administered at doses of 150 µmol/kg and 300 µmol/kg, a further increase in hypnosis time was observed (439.8 ± 82.7 s and 1017.0 ± 313.0 s, respectively (Figure 5).

**Figure 6:**
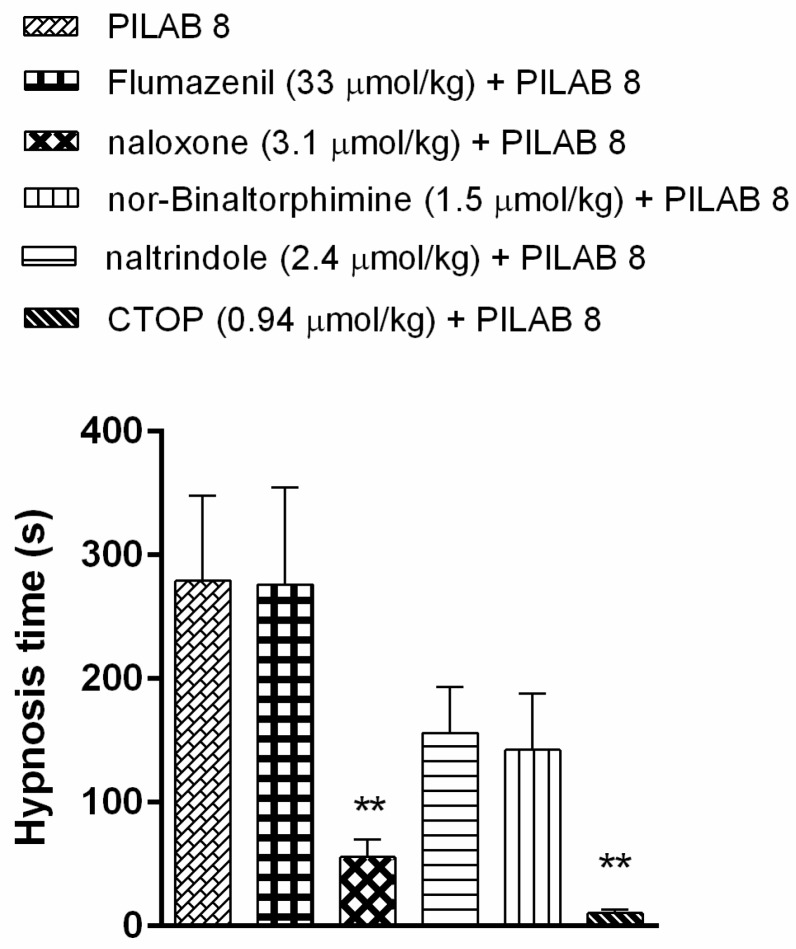
Effects of **PILAB 8** (100 μmol/kg) on hypnosis time following pre-treatment with flumazenil (33 μmol/kg), naloxone (3.1 μmol/kg), nor-binaltorphimine (1.5 μmol/kg), naltrindole (2.4 μmol/kg), or CTOP (0.94 μmol/kg). Time intervals between loss and recovery of the righting reflex were recorded. Data are expressed as the mean ± SEM (*n* = 10). ** *p* < 0.01 compared to **PILAB 8** (100 μmol/kg i.v.), one-way ANOVA followed by Dunnett's multiple comparison test for parametric measures

To evaluate the mechanism mediating the observed increase in hypnosis time following administration of **PILAB 8**, mice were pre-treated with naloxone and other specific opioid pathway antagonists. The hypnosis time associated with naxolone decreased to 55.7 ± 14.3 s, while the administration of naltrindole, nor-binaltorphimine, and CTOP reduced the hypnosis times to 156.1 ± 37.0 s, 142.6 ± 45.1 s and 10.6 ± 2.8 s, respectively (Figure 6).

### 2.3. Effect of **PILAB 8** on Locomotor Activity and Performance in the Moto Coordination (Rotarod Test)

**Figure 7:**
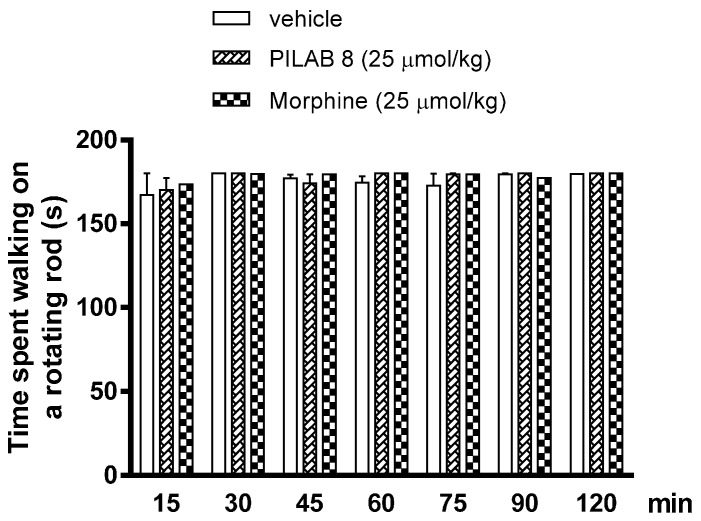
Effects of vehicle versus **PILAB 8** on motor coordination. Mice received an i.p. injection of vehicle or **PILAB 8** (100 μmol/kg) and then underwent a rotarod test 15, 30, 45, 60, 75, 90, and 120 min later. Data are expressed as the mean time spent walking on the rotating rod ± SEM (*n* = 10) and were analyzed with the Kruskal-Wallis test followed by Dunnett’s multiple comparison test for parametric measures.

None significant impairment in motor activity was detected following an i.p. injection of **PILAB 8** (25 µmol/kg) in the rotarod test compared with the mice that received an i.p. injection of vehicle (Figure 7).

### 2.4. Effect of **PILAB 8** on the Anxiolytic Response

**Figure 8:**
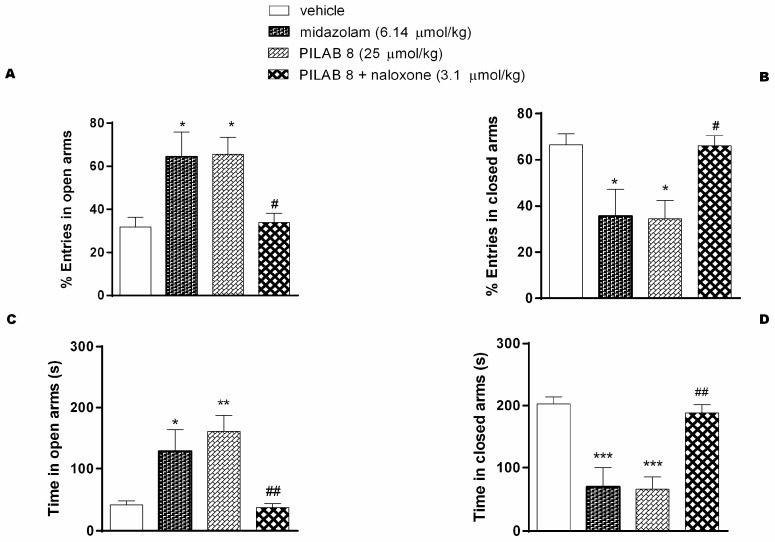
Effects of **PILAB 8** (25 μmol/kg) or midazolam (6.15 μmol/kg) with or without prior administration of naloxone (3.1 μmol/kg). The percentage of entries into the open arms (**A**) and the closed arms (**B**) of the EPM over a 5 min interval are presented. * *p* < 0.05, ** *p* < 0.01, and *** *p* < 0.001 vs. vehicle; ^##^ *p* < 0.01 vs. **PILAB 8**. The time spent in the open arms (**C**) and the closed arms (**D**) of the EPM over a 5 min interval. * *p* < 0.05, ** *p* < 0.01 and *** *p* < 0.001 vs. vehicle; ^#^ *p* < 0.05, ^##^ *p* < 0.01 vs. **PILAB 8**.

Figure 8 shows the percentage of the number of entries into the open and closed arms on the EPM by the various groups. The percentage of entries into the open arms by the mice that were treated with **PILAB 8** and midazolam increased from 32 ± 4% (value for the control group treated with vehicle) to 64 ± 11% and 65 ± 7%, respectively. For entries into the closed arms, the percentage values decreased from 66 ± 5 (for the control group treated with vehicle) to 35 ± 11% and 34 ± 7% respectively.

Treatment with **PILAB 8** and midazolam also increased the time spent in the open arms from 41.3 ± 6 s (vehicle group) to 161 ± 26 s and 129 ± 35 s, respectively. Conversely, the time spent in the closed arms decreased from 203 ± 11 s to 66 ± 19 s and 70 ± 30 s, respectively. When the mice were pre-treated with naloxone and then were treated with **PILAB 8**, the percentages for open and closed arm entries were 34 ± 4% and 66 ± 4%, respectively. The time spent in the open and closed arms were 37 ± 7 s and 188 ± 13 s, respectively.

### 2.5. Effect of **PILAB 8** on Formalin-Induced Nociception Response

**Figure 9:**
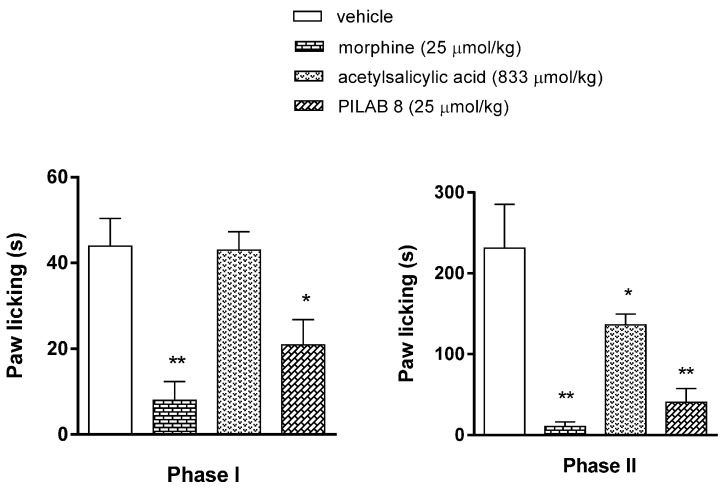
Evaluation of the antinociceptive activity of **PILAB 8** (25 μmol/kg i.p.), morphine (25 μmol/kg i.p.) and acetylsalicylic acid (833 μmol/kg i.p.) in the formalin test. Intraperitoneal injections each substance were performed 30 min prior to the injection of formalin and subsequently observed during 30 min. Data are expressed as the mean time of reactivity (time spent licking) ± SEM (*n* = 10). * *p* < 0.05 and ** *p* < 0.01 vs vehicle-treated group, one-way ANOVA followed by Dunnett’s test.

An i.pl. injection of formalin (20 µL) was performed to provoke a classical nociceptive response (time of flinching, lifting, licking, shaking, biting behavior) in two phases. In the neurogenic phase, mice that were administered **PILAB 8** or morphine exhibited reduced formalin responsiveness from 44 ± 6 s (for the vehicle group) to 21 ± 5 s and 8 ± 4 s, respectively. In contrast, administration of acetylsalicylic acid did not attenuate formalin responsivity. In the inflammatory phase, the animals treated with **PILAB 8**, morphine, or acetylsalicylic acid all exhibited a decrease in formalin responsiveness from 231 ± 54 s (for the vehicle group) to 40 ± 17 s, 10 ± 6 s and 93 ± 19 s, respectively (Figure 9).

### 2.6. Effect of **PILAB 8** on Hot Plate-Induced Nociception Response

When mice received an i.p. injection of **PILAB 8** (25 µmol/kg) prior to a hot plate test, the mice exhibited a antinociceptive activity percentage of (38 ± 8%) 70 min later. Compared with the animals that received vehicle (6 ± 3%), this increase in latency response to thermal stimuli was significant.

**Figure 10:**
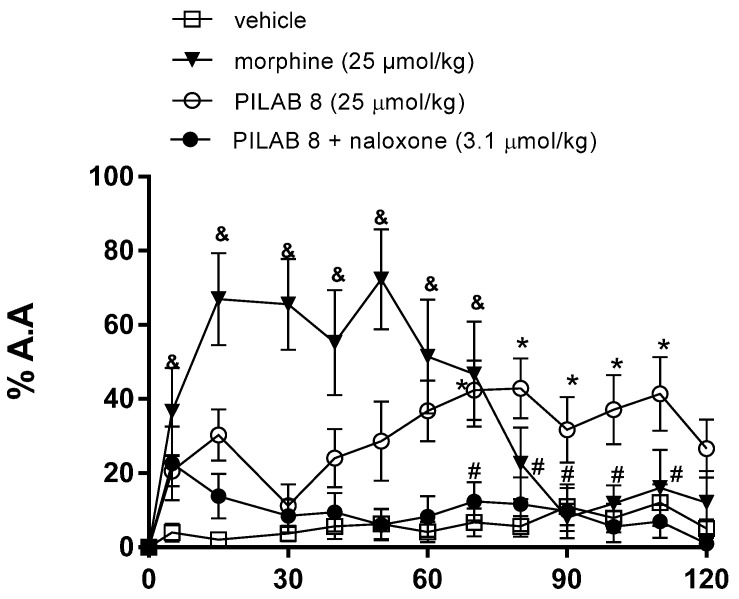
Effects of the intraperitoneal injection of vehicle, **PILAB 8** (25 μmol/kg), morphine (25 μmol/kg) and pretreatment with naloxone (3,1 μmol/kg) in animals received **PILAB 8** (i.p.) in a hot plate test. Data are expressed as the mean ± SEM (*n* = 8–10). * *p* < 0.05 and ^&^ *p* < 0.05 vs. vehicle; ^#^ *p* < 0.05 vs. **PILAB 8**, one-way ANOVA followed by Dunnett’s test.

Possible involvement of the opioid system in mediating the antinociceptive effect of **PILAB 8** was subsequently examined. When mice were pre-treated with naloxone (3.1 µmol/kg, i.p.) for 15 min prior to an i.p. injection of **PILAB 8**, antinociception was significantly reduced to 12 ± 5% at the 70 min time point for the hot plate test (Figure 10).

## 3. Discussion

Various isatin-type 5′-4-alkyl/aryl-1*H*-1,2,3-triazoles **PILAB 1**–**12** were initially evaluated in the pentobarbital-induced sleep assay. All of the isatin-triazole derivatives significantly increased the duration of hypnosis. **PILAB 6**, **PILAB 8** and **PILAB 9** were particularly effective, and all of these contain a linear alkyl chain linked to the triazole ring. These results suggest that the apolar portion present in the triazole plays a key role in facilitating the movement of this compound across the blood brain barrier. Moreover, after an i.v. injection of **PILAB 8**, a hypnotic-like profile approximately 3 times greater than that achieved with **PILAB 6** and **PILAB 7** was observed. Compound **PILAB 9**, containing a propyl group attached to the triazole ring, also induced a hypnotic-like profile, although it was not greater than the hypnotic profiles of **PILAB 6** (R′ = butyl) and **PILAB 8** (R′ = pentyl). Those results indicated that the size of the alkyl chain is an important factor to the modulation of the activity. In contrast, compounds **PILAB 1** and **PILAB 10**, containing a phenyl and cyclohex-1-en-1-yl group, respectively, did not produce important effects. Similar results were observed with polar compounds like **PILAB 2** (R′ = hydroxymethyl), PILAB 3 (R′ = 2-hydroxy-propan-2-yl) and **PILAB 5** (R′ = 1-hydroxycyclohexyl).

The hypnotic profile of **PILAB 8** was of particular interest and subsequent pretreatment experiments with the non-selective opioid antagonist, naloxone, and the µ-opioid selective antagonist, CTOP, were found to prevent the hypnosis induced by **PILAB 8**.

It is hypothesized that **PILAB 8** could bind and activate the µ-opioid receptor, which represents a coupled Gi protein. To date, agonist µ-opioid receptors induce analgesia for relief of some of the most chronic types of pain. However, activation of these receptors may produce adverse effects such as respiratory depression, sedation, addiction, and tolerance, and these side effects limit their clinical use [22].

In the present study, when **PILAB 8** was administered at a dose of 25 µmol/kg via an i.p. injection, neither sedation nor locomotor activity alterations were observed. Intraperitoneal administration of **PILAB 8** did not result in locomotor activity changes, ensuring continuity for assessments of other behavioral assessments since changes might reduce the behavioral response, thereby resulting in a false positive effect.

The anxiolytic profile of **PILAB 8** (25 µmol/kg, i.p.) was examined with an EPM test to investigate both physiological and pharmacological behavior [23]. When animals are less anxious they tend to enter the open arms of the device and stay there longer. Meanwhile, an anxiogenic substance produces the opposite effects [24,25,26,27,28]. The opioid pathway plays an important role in the modulation of anxiety, and activation of this pathway has been hypothesized to mediate anxiolytic responses [29]. For example, when the µ-opioid receptor agonist, endomorphine 1, was administered intracerebroventricularly into mice, an anxiolytic effect was observed in the EPM test [29,30,31,32]. Based on these findings and the results of the EPM assays conducted in the present study, it appears that the anxiolytic effect of **PILAB 8** is mediated via µ-opioid receptors.

Injection of formalin induced initially a neurogenic phase followed by a inflammatory-induced pain [33,34,35,36]. The early phase is consequent to a stimulation of nociceptors, activation of C-fiber afferents which resulted in increased release of glutamate and aspartate into the dorsal horn [34,37,38]. The late phase is due to a local inflammatory reaction, which promotes the release of prostaglandins, bradykinin, serotonin and histamine [34,39]. Generally, drugs such as opioids inhibit both phases of the formalin test [40] However, the peripheral action of drugs such as non-narcotic agents have been observed to improve the nociceptive response to formalin in the second phase, while the initial antinociception phase remains unaffected [34]. Our results demonstrated that **PILAB 8** inhibited both phases of the formalin-induced nociception test, thus a noxious thermal stimulus induced by a hot plate was used to investigate the analgesic drug action [33,41]. At a dose of 25 µmol/kg, **PILAB 8** increased the percentage of the antinociceptive response and this response reverted when the mice were pretreated with naloxone, an opioid antagonist. In the present study, **PILAB 8** exhibited antinociceptive action. It was previously reported that a subset of isatin analogs exhibit antinociceptive effects in both chemical and thermal models of nociception, and the mechanism of action may involve the opioid pathway [42]. Primary afferent nociceptive fibers express µ-opioid receptors, and the majority of these receptors are localized to the periphery of the fibers. Moreover, activation of these µ-opioid receptors has been hypothesized to produce antinociceptive effect [43,44,45]. It is hypothesized that **PILAB 8**, a µ-opioid agonist, may provide beneficial effects in animal models of pain activating the opioid system to induce an antinociception mechanism.

## 4. Material and Methods

### 4.1. Synthesis of Hybrid Triazole-Isatin Derivatives

Derivatives of 5′-(4-akyl/aryl-1*H*-1,2,3-triazole)-isatin were obtained by treating 5-azido-spiro[1,3-dioxolane-2,3′-indol]-2′(1′*H*)-one with various alkynes under acidic conditions followed by the application of ultrasound irradiation as described by Silva et al. [20,21]. The twelve 5′-(4-akyl/aryl-1*H*-1,2,3-triazole)-isatin derivatives **PILAB 1**–**12**, as well as the precursors, isatin and 5-azido-spiro[1,3-dioxolane-2,3′-indol]-2′(1′*H*)-one (**1**), were evaluated for hypnotic-sedative activity (Table S1).

### 4.2. Analysis of the Purity of the Compounds Evaluated by HPLC (High Performance Liquid Chromatography)

The HPLC analysis was conducted on a Shimadzu LC20AT system (Shimadzu, Kyoto, Japan). The Shimadzu Lab solutions software was used for data acquisition. Acetonitrile/methanol (5:95 *v*/*v*) was used as the mobile phase with a 150  ×  4.6-mm Eclipse Plus C18 column. The flow rate was 1 mL/min and the injection volume was 1 μL. The wavelength of detection used was 280 nm. The purities of the compounds are shown in Table S2.

### 4.3. Animals

The experimental protocols used in the present study were approved by the Animal Care and Use Committee of the Universidade Federal do Rio de Janeiro, Brazil (CEUA/UFRJ DFBCICB068). Briefly, male Swiss mice (25–35 g) were kept in polypropylene boxes containing sawdust and were maintained under controlled temperature (21 ± 1 °C) and humidity (60%) with a 12-h light/dark cycle (lights on at 6 a.m.). Food and water were provided *ad libitum*. Animals were moved into the experiment room at least 30 min before the start of the tests in order for the mice to adapt to the new environment. The mice were randomly divided into control and treatment groups (*n* = 6–10).

### 4.4. Drugs

The **PILAB** compounds, azide, and triazoles were kindly donated by the Laboratório de Produtos Naturais e Transformações Químicas (IQ-UFRJ, Rio de Janeiro, Brazil). Acetylsalicylic acid (Sigma, Saint Louis, MO, USA), morphine, flumazenil, midazolam, and diazepam (Cristália, Itapira, Brazil) were freshly prepared in dimethyl sulfoxide (DMSO, Cristália) minutes prior to the experiments. Naloxone (Cristália), formaldehyde (Isofar, Duque de Caxias, Brazil), nor-binalthophimine, naltrindole, and CTOP and pentobarbital sodium salt (Tocris Bioscience, Minneapolis, MN, USA) were dissolved in distilled water.

### 4.5. Pentobarbital-Induced Sleep Test

The hypnotic effect of the compounds investigated was examined in a pentobarbital-induced sleep test as previously described [46]. Briefly, PILABs (100 µmol/kg) were administered via an intraperitoneal (i.p.) injection 10 min prior to the intravenous (i.v.) administration of pentobarbital sodium (25 mg/kg). Hypnosis time was considered to be the difference between the time of loss of the postural reflex and the time of its recovery. A control group received an intraperitoneal (i.p.) injection of DMSO and an i.v. injection of pentobarbital.

The time of hypnosis was further examined with the administration of vehicle and **PILAB 4**, **6**, **7**, **8**, **9**, **11** and **12** (100 µmol/kg i.v.) into tail of the animal. **PILAB 8** was also administered at increasing doses to evaluate response dose in relation to hypnosis time. To investigate the mechanisms mediating the induction of hypnosis, mice were pre-treated with an i.p. injection of: flumazenil (33 µmol/kg), a benzodiazepine antagonist [47]; naloxone (3.1 µmol/kg), a non-selective opioid receptor antagonist [48]; nor-binaltorphimine (1.5 µmol/kg), a selective kappa opioid receptor antagonist); or naltrindole (2.4 µmol/kg), a selective delta opioid receptor antagonist [49]. All of these antagonists were administered 15 min prior to the administration of **PILAB 8** (i.v.), except for CTOP (0.94 µmol/kg), a selective mu opioid receptor antagonist, which was administered 15 min prior to **PILAB 8** [50].

### 4.6. Motor Coordination (Rotarod Test)

Dunham and Miya described a method for detecting motor harm in response to substances such as skeletal muscle relaxants or CNS depressants [51,52]. A rotarod treadmill (Insight, Model EFF 411, Ribeirão Preto, Brazil) consisting of a bar with a diameter of 2.7 cm and height of 40 cm was subdivided into four compartments by using disks 25 cm in diameter that rotate at 8 revolutions per minute (rpm). Male Swiss mice (20–25 g) were placed on the apparatus in three training sessions 24 h prior to testing as previously described [53]. The mice that could not sustain themselves on the apparatus for more than 90 s were excluded. Motor performance was evaluated based on the time spent walking on a rotating rod (8 rpm) over a 3 min interval at various time points after an i.p. injection (15, 30, 45, 60, 75, 90 and 120 min) of vehicle, **PILAB 8** or morphine (25 µmol/kg).

### 4.7. Anxiolytic Activity (Elevated Plus-Maze (EPM) Test)

An LE 846 apparatus (Panlab, Barcelona, Spain) was used which contains two open arms and two closed arms connected by a central platform. Both arms are elevated to a height of 50 cm from the floor. An animal’s position is registered by eight photoelectric cells that are arranged in each arm so that nine sectors are defined. Data were recorded with the Mazesoft-4 Software. Male Swiss mice were treated with an i.p. injection of vehicle, **PILAB 8** (25 µmol/kg), or midazolam (6.14 μmol/kg) 10 min before being placed on the central platform of the maze facing an open arm. The percentage of entries into the open and closed arms, as well as the time spent in each set of arms, were counted during a 5-min test period. 

### 4.8. Antinociceptive Activity Evaluation of **PILAB 8**

#### 4.8.1. Formalin Test

A formalin test was performed based on a protocol previously described for the quantitative study of antinociceptive effects [54]. Briefly, an intraplantar administration (i.pl.) of formalin was performed to provoke two phases of nociception behavior. The first phase (0–5 min after the injection) is referred to as the neurogenic phase. This phase is followed by a short quiescent period (5–15 min) that precedes the second phase (15–30 min after the injection) that includes an inflammatory response. For this study, formalin (20 μL, 2.5%) was administered via an i.pl. injection into the right hind paw of each animal 30 min after an i.p. injection was made of vehicle, acetyl salicylic acid (833 µmol/kg), morphine (25 µmol/kg), or **PILAB 8** (25 µmol/kg). The total time spent by each animal licking in the injected paw was then observed for 30 min.

#### 4.8.2. Hot Plat Test

Central analgesic activity was evaluated in a hot plate test. Briefly, mice were placed onto a hot plate maintained at 52 ± 1 °C (LE 7406, Letica, Letica Scientific Instruments, Barcelona, Spain). The latency of their nociceptive threshold was recorded according to the time until licking or shaking of one of their paws or jumping was observed. Maximal permanence of the animals on the hot plate was 35 s to avoid damage to the paws. Animals received an i.p. injection of vehicle, **PILAB 8** (25 µmol/kg), or morphine (25 µmol/kg). Involvement of the opioid pathway was investigated by administering naloxone (3.1 µmol/kg i.p.) 15 min prior to the administration of **PILAB 8**. Antinociceptive activity (AA%) was calculated using Equation (1):

%AA = (postdrug latency) − (predrug latency) × 100% (35 s) − (predrug latency)(1)

### 4.9. Statistical Analysis

Data are expressed as the mean ± standard error of the mean (SEM) and were analyzed using one-way analysis of variance (ANOVA) followed by Dunnett’s multiple comparison test. GraphPad Prism, version 6.0 (GraphPad Software Inc., San Diego, CA, USA), was used to perform the statistical analyses and differences with a *p* > 0.05 were considered significant.

## 5. Conclusions

In conclusion, the results of the present study provide evidence that the triazole scaffold potentiates the activity of isatin ketals and an apolar substituent attached to this ring increases the effects on the CNS. **PILAB 8**, bearing the longest alkyl chain among the tested compounds, showed the best hypnotic profile. This compound was selected for subsequent experiments, indicating that can activate µ-opioid receptors to provoke antinociceptive effect without morphine-like side effects. In addition, **PILAB 8** was found to effectively reverse anxiety independent of a pain response.

## Supplementary Materials

Supplementary materials are available online.
