# Supplementary material for: The Hypnotic, Anxiolytic, and Antinociceptive Profile of a Novel µ-Opioid Agonist
Source: Molecules. 2017 May 16;22(5):800. doi: 10.3390/molecules22050800 (PMC6154531; doi:10.3390/molecules22050800)
Supplement: Supplementary file 1 [file molecules-22-00800-s001.pdf]

Supplementary material: The anxiolytic, anti-inflammatory, and antinociceptive profile of a novel μ-opioid agonist

Table S1. Chemical characteristics of evaluated compounds

Compound	MP[a] (°C)	HR-ESI-MS $[M+H]^{+\cdot}$; IR (KBr)[b]	^{1}H NMR; ^{13}C NMR[c]
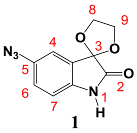 **1**	-	HRMS: Found 255.0493, $C_{10}H_8N_4NaO_3^{+\cdot}$ calculated: 255.0489;	NMR ^{1}H (DMSO-d_6, 200 MHz) δ 10.52 (sl, 1H, NH); 7.11–7.07 (m, 2H, H4 and H6); 6.87 (d, *J* = 8 Hz, 1H, H7), 4.38–4.23 (m, 4H, H8 and H9); NMR ^{13}C (DMSO-d_6, 50 MHz) δ 174.73 (Cq); 140.47 (Cq); 134.42 (Cq); 127.07 (Cq); 122.73 (CH); 116.65 (CH); 112.32 (CH); 101,97 (Cq); 66.19 (CH_2).
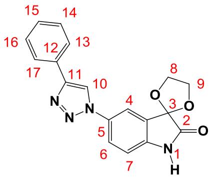 **PILAB 1**	230	HRMS: Found: 335.1134, $C_{18}H_{15}N_4O_3^{+}$ calculated: 335.1139; IR (KBr)/cm^{-1}: 3291, 1736, 1637, 1508, 1470, 1292, 1213, 1077, 762, 553 and 507.	NMR ^{1}H (DMSO-d_6, 400 MHz) δ 10.80 (s, 1H, NH); 9.25 (s, 1H, H10); 7.95–7.93 (m, 4H); 7.51–7.47 (m, 2H); 7.37 (t, *J* = 8 Hz, 1H); 7.08 (d, *J* = 8 Hz, 1H, H7); 4.42–4.32 (m, 4H, H8 and H9); NMR ^{13}C (DMSO-d_6, 100 MHz) δ 174.77 (Cq); 147.62 (Cq); 143.41 (Cq); 132.40 (Cq); 130.72 (Cq); 129.43 (CH); 128.60 (CH); 126.50 (Cq); 125.73 (CH); 124.26 (Cq); 120.11 (CH); 117.82 (CH); 111.98 (CH); 111.95 (CH); 101.77 (Cq); 66.23 (CH_2).
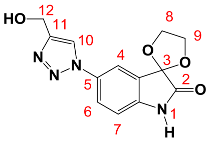 **PILAB 2**	225	HRMS: Found: 289.0936, $C_{13}H_{13}N_4O_4^{+}$ calculated: 289.0931; IR (KBr)/cm^{-1}: 3443, 3143, 2968, 1727, 1635, 1508, 1473, 1289, 1149, 994, 844 and 547.	NMR ^{1}H (DMSO-d_6, 400 MHz) δ 10.74 (s, 1H, NH); 8.64 (s, 1H, H10); 7.89–7.87 (m, 2H, H4 and H6); 7,02 (d, *J* = 8 Hz, 1H, H7); 5.34 (t, *J* = 8Hz, OH); 4.61 (d, *J* = 8 Hz, 2H, H12); 4.39–4.30 (m, 4H, H8 and H9); NMR ^{13}C (DMSO-d_6, 100 MHz) δ 174,72 (Cq); 149.41 (Cq); 143.13 (Cq); 132.52 (Cq); 126.67 (Cq); 124.03 (CH); 121.43 (CH); 117.74 (CH); 111.84 (CH); 101.74 (Cq); 66.23 (CH_2); 55.40 (CH_2).
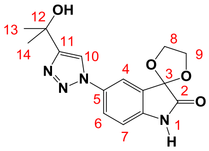 **PILAB 3**	132	HRMS: Found: 317.1238, $C_{15}H_{17}N_4O_4^{+}$ calculated: 317.1244; IR (KBr)/cm^{-1}: 3461,2979, 1727, 1631, 1508, 1286, 1158, 1080, 837, 723 and 554.	NMR ^{1}H (DMSO-d_6, 400 MHz) δ 10.74 (sl, 1H, NH); 8.56 (s, 1H, H10); 7.90–7.88 (m, 2H, H4 and H6); 7.02 (d, *J* = 12 Hz, 1H, H7); 5.23 (sl, 1H, OH); 4,39–4,30 (m, 4H, H8 and H9); 1.53 (s, 6H, H13 e H14); NMR ^{13}C (DMSO-d_6, 100 MHz) δ 174.73 (Cq); 157.16 (Cq), 143.01(Cq); 132.60 (Cq); 132.60 (CH); 126.67 (Cq); 119,30 (CH); 117.64 (CH); 111.84 (CH); 101.75 (CH);

			67.39 (Cq); 66.24 (CH_2); 30.97 (CH_2).
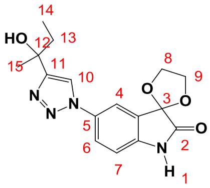 PILAB 4	92	HRMS: Found 331.1407, $C_{16}H_{19}N_4O_4^+$ calculated 331.1401; IR (KBr)/cm^{-1}: 3485, 2973, 1734, 1631, 1504, 1293, 1215, 1143, 1052, 754 and 545.	NMR ^{1}H (DMSO-d$_6$, 400 MHz) δ 10.72 (sl, 1H, NH); 8.53 (sl, 1H, H10); 7.90–7.88 (m, 2H, H4 and H6); 7.01 (d, *J* = 8 Hz, 1H, H7); 5.07 (s, 1H, OH); 4.38–4.30 (m, 4H, H8 e H9); 1.79 (quart, *J* = 8 Hz, 2H, H12); 1.49 (s, 1H, H14); 0.79 (t, *J* = 8 Hz, 3H, H13); NMR ^{13}C (DMSO-d$_6$, 100 MHz) δ 174,72 (Cq); 156.18 (Cq); 142.95 (Cq); 132.60 (Cq); 126.70 (Cq); 123.82 (CH); 119.96 (CH); 119.94 (CH), 117.58 (CH); 111.82 (CH); 101.75 (Cq); 70.14 (Cq); 66.26 (CH_2); 35.74 (CH3); 28.47 (CH_2); 8.79 (CH_3).
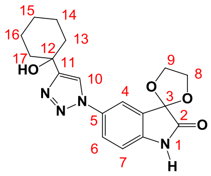 PILAB 5	188	HRMS: Found 357.1561, $C_{18}H_{21}N_4O_4^+$ calculated 357.1557; IR (KBr)/cm^{-1}: 3268, 3104, 2931, 1754, 1632, 1498, 1279, 970, 896 and 605.	NMR ^{1}H (DMSO-d$_6$, 400 MHz) δ 10.72 (sl, 1H, NH); 8.58 (s, 1H, H10); 7.90–7.88 (m, 2H, H4 and H6); 7.01 (d, *J* = 12 Hz, 1H, H7); 4,97 (s, 1H, OH), 4,39–4,30 (m, 4H, H8 and H9); 4.05–4.00 (quart, *J* = 8 Hz, 2H), 1,98–1,91 (m, 4H), 1.79–1.68 (m, 4H), 1,17 (t, *J* = 8 Hz, 2H); NMR ^{13}C (DMSO-d$_6$, 100 MHz) δ 174.74 (Cq); 156.98 (Cq); 143.00 (Cq); 132.63 (Cq); 126.67 (Cq), 123.86 (CH). 119.79 (CH); 119.77 (CH), 117.59 (CH); 111.83 (CH); 101.77 (Cq); 68.37 (CH_2); 66.25 (CH_2); 25.68 (CH_2); 22.18 (CH_2); 21.19 (CH_2); 14.52 (CH_2).
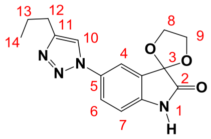 PILAB 6	181	HRMS: Found 301.1289, $C_{15}H_{17}N_4O_3^+$ calculated 301.1295; IR (KBr)/cm^{-1}: 3315, 3076, 2960, 1748, 1633, 1507, 1223, 1081, 947, 838 and 545.	NMR ^{1}H (DMSO-$_{d6}$, 400 MHz) δ 10.73 (sl, 1H, NH); 8.52 (s, 1H, H10); 7.86–7.84 (m, 2H, H4 and H6); 7.02 (d, *J* = 12 Hz, 1H, H7); 4.39–4.30 (m, 4H, H8 and H9); 2.66 (t, *J* = 8 Hz, 2H, H12); 1,67 (sext, *J* = 8Hz, 2H, H13); 0.94 (t, *J* = 8 Hz, 3H, H14); NMR ^{13}C (DMSO-$_{d6}$, 100 MHz) δ 174.74 (Cq); 148.30 (Cq); 143.02 (Cq); 132.62 (Cq); 126.55 (Cq); 123.91 (CH); 120.61 (CH); 117.60 (CH); 111.83 (CH); 101.77 (Cq); 66.22 (CH_2); 27.50 (CH_2); 22.51 (CH_2); 14.07 (CH_3).
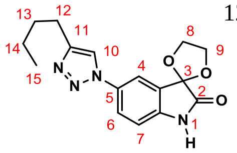 PILAB 7	127	HRMS: Found: 315.1455. $C_{16}H_{19}N_4O_3^{+\cdot}$ calculated 315.1452; IR (KBr)/cm^{-1}: 3316, 2927, 1751, 1633, 1506, 1221, 1091, 1034, 947 and 838.	NMR ^{1}H (DMSO-$_{d6}$, 400 MHz) δ; 10.72 (s, 1H, NH); 8.53 (s, 1H, H10); 7.86–7.84 (m, 2H, H4 and H6); 6,99 (d, *J* = 8 Hz, 1H, H7); 4.37–4.28 (m, 4H, H8 and H9); 2.68 (t, *J* = 8 Hz, 2H, H12); 1,63 (quint, *J* = 8 Hz, 2H, H13); 1.36 (sext, J = 8Hz, 2H, H14); 0,91 (t, *J* = 8Hz, 3H, H15); NMR ^{13}C (DMSO-$_{d6}$, 100 MHz) δ 174,74 (Cq); 143.00 (Cq); 132,63 (Cq); 126,55 (Cq); 123,89 (CH); 120.63 (CH); 117,57 (CH); 111,83 (CH);

			109,99 (Cq); 101.76 (Cq); 66.21 (CH_2); 31,32 (CH_2); 25,13 (CH_2); 22.11 (CH_2); 14.15 (CH_3).
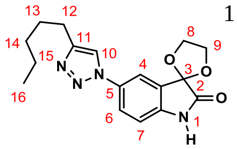 PILAB 8	112	HRMS: Found 329.1615, $C_{17}H_{21}N_4O_3^+$ calculated 329.1608; IR (KBr)/cm^{-1}: 3336, 3111, 2952, 1747, 1632, 1505, 1282, 944, 751 and 546.	NMR ^{1}H (DMSO-$_{d6}$, 400 MHz) δ 10.74 (sl, 1H, NH); 8.50 (s, 1H, H10); 7.85–7.83 (m, 2H, H4 and H6); 7.02 (d, *J* = 8 Hz, 1H, H7); 4.38–4.29 (m, 4H, H8 and H9); 2.66 (t, *J* = 8 Hz, 2H, H12); 1.64 (quint, J = 8Hz, 2H, H13); 1.32–1.31 (m, 4H, H14 and H15); 0.86 (t, *J* = 4 Hz, 3H, H16); NMR ^{13}C (DMSO-$_{d6}$, 100 MHz) δ 174.77 (Cq); 148,51 (Cq); 143.00 (Cq); 132.61 (Cq); 126.57 (Cq); 123.92 (CH); 120.52 (CH); 117.58 (CH); 111.87 (CH); 101.77 (Cq); 66.23 (CH_2); 31.22 (CH_2); 28.88 (CH_2); 25.39 (CH_2); 22.31 (CH_2); 14.31(CH_3).
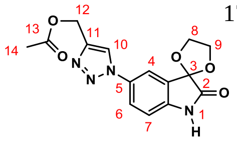 PILAB 9	171	HRMS: Found 331.1026, $C_{15}H_{15}N_4O_5^+$ calculated 331.1037; IR (KBr)/cm^{-1}: 3154, 2899, 1748, 1632, 1507, 1217, 1038, 998, 730 and 543.	NMR ^{1}H (DMSO-$_{d6}$, 400 MHz) δ 10.74 (s, 1H, NH); 8.79 (s, 1H, H10); 7.88–7.86 (m, 2H, H4 and H6); 7.03 (d, *J* = 8 Hz, 1H, H7); 5.20 (s, 2H, H12); 4.39–4.31 (m, 4H, H8 and H9); 2.06 (sl, 3H, H14); NMR ^{13}C (DMSO-$_{d6}$, 100 MHz) δ 174.72 (Cq); 170.53 (Cq); 143.45 (Cq); 143.43 (Cq); 132.26 (Cq); 126.66 (Cq); 124.39 (CH); 123.36 (CH); 118.62 (CH); 111.87 (CH); 101.73 (Cq); 66.25 (CH_2); 57.40 (CH_2); 21.05 (CH_3).
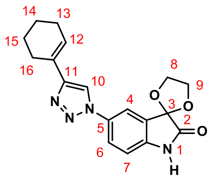 PILAB 10	227	HRMS: Found 339.1447, $C_{18}H_{19}N_4O_3^+$ calculated 339.1452; IR (KBr)/cm^{-1}: 3197, 3163, 2935, 1742, 1628, 1505, 1277, 1056, 954, 777 and 728.	NMR ^{1}H (DMSO-$_{d6}$, 400 MHz) δ 10.75 (s, 1H, NH); 8,73 (s, 1H, H10); 7.89–7.87 (m, 2H, H4, H6); 7.03 (d, *J* = 8 Hz, 1H, H7); 6.51 (sl, 1H, H11); 4.40–4.30 (m, 4H, H8 and H9); 2.38 (m, 2H); 2.17 (m, 2H); 1.66 (m, 4H); NMR ^{13}C (DMSO-$_{d6}$, 100 MHz) δ 174.74 (Cq); 149.33 (Cq); 143.13 (Cq); 132.51 (Cq); 127.60 (Cq); 126.53 (Cq); 124.69 (CH); 123.92 (CH); 118.44 (CH); 117.56 (CH); 111.86 (CH); 101.79 (Cq); 66.21 (CH2); 26.24 (CH_2); 25.16 (CH_2); 22.44 (CH_2); 22.29 (CH_2).
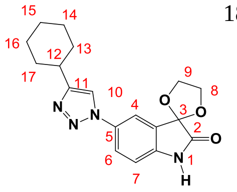 PILAB 11	180	HRMS: Found 341.1602, $C_{18}H_{21}N_4O_3^+$ calculated 341.1608; IR (KBr)/cm^{-1}: 3313, 3148, 2924, 1755, 1630, 1267, 1218, 1063, 996, 825, 726 and 550.	NMR ^{1}H (DMSO-$_{d6}$, 400 MHz) δ 10.71 (s, 1H, NH); 8.50 (s, 1H, H10); 7.86–7.84 (m, 2H, H4 and H6); 7.01 (d, *J* = 8 Hz, 1H, H7); 4.39–4.30 (m, 4H, H8 and H9); 2.72 (quint, 1H, H12); 2.02–2.00 (m, 2H); 1.91 (sl, 3H); 1.77–1.74 (m, 3H); 1.48–1.36 (m, 4H); NMR ^{13}C (DMSO-$_{d6}$, 100 MHz) δ 174.74 (Cq); 172.47 (Cq); 153,64 (Cq); 142.98 (Cq); 132,66 (Cq); 126.55 (Cq);

			123.85(CH); 119.39 (CH); 117,56 (CH); 111.83 (CH); 109.99; 101.77; 66.21(CH_2); 35.04 (CH); 32.87 (CH_2); 25.96 (CH_2); 25.96 (CH_2); 21.48 (CH_2).
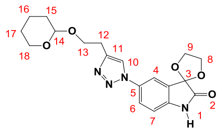 PILAB 12	121	HRMS: Found 387.1242, $C_{15}H_{17}N_4O_4^+$ calculated 387.1244; IR (KBr)/cm^{-1}: 3211, 2945, 1747, 1635, 1509, 1271, 1192, 1090, 988, 735 and 544.	NMR ^{1}H (DMSO-$_{d6}$, 400 MHz) δ 10.73 (s, 1H, NH); 8.53 (s, 1H, H10); 7.86–7.84 (m, 2H, H4, H6); 7.02 (d, *J* = 8 Hz, 1H, H7); 4.55–4.53 (m, 1H, H14); 4.39–4.30 (m, 4H, H8 and H9); 3.76–3.66 (m, 3H); 2.76 (t, 2H); 1.91 (quint, 2H), 1.72–1.59 (m, 4H); NMR ^{13}C (DMSO-$_{d6}$, 100 MHz) δ 174.73 (Cq); 148.07 (Cq); 143.05 (Cq); 132.60 (Cq); 126.56 (Cq); 123.89 (CH); 120.68 (CH); 117.60 (CH); 111.83 (CH); 109.99 (CH); 101.77 (Cq); 98.44 (CH_2); 66.21 (CH_2); 61.75 (CH); 30.76 (CH_2); 29.36 (CH_2); 25.49 (CH_2); 22.38; 19.64 (CH_2).

[a] MP – melting point

[b] HR-ESI-MS - High-*resolution electrospray* ionization mass *spectrometry*

[c] ^{1}H NMR - *Hydrogen-1 nuclear magnetic resonance ; ^{13}C NMR - Carbon-13 nuclear magnetic resonance*

Analysis of the purity of the compounds evaluated by HPLC (High performance liquid chromatography)

The HPLC analysis was conducted on Shimadzu LC20AT. Shimadzu Lab solutions software was used for data acquisition. Acetonitrile/methanol (5:95 *v*/*v*) was used as the mobile phase with a 150 × 4.6-mm Eclipse Plus C18 column. The flow rate was 1 mL/min and the injection volume was 1 μL. The wavelength of detection used was 280 nm. The purities of the compounds are shown in Table S2, and the chromatograms are arranged on the following pages.

Table S2. Retention time (min) and purity (%) of **PILAB1** – **PILAB 12**

	Retention Time (min)	Purity (%)
PILAB1	18.231	98.94
PILAB2	11.661	93.90
PILAB3	14.823	98.47
PILAB5	19.055	99.40
PILAB6	17.641	97.94
PILAB7	16.147	99.39
PILAB8	13.353	99.78
PILAB9	3.785	99.88
PILAB10	19.078	99.35
PILAB12	19.039	96.64

Chromatograms

PILAB1

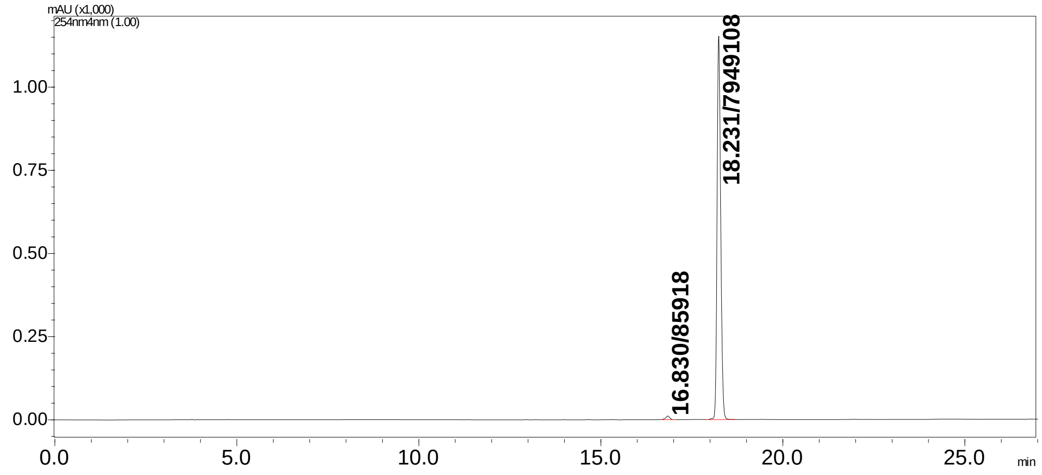

PILAB 2

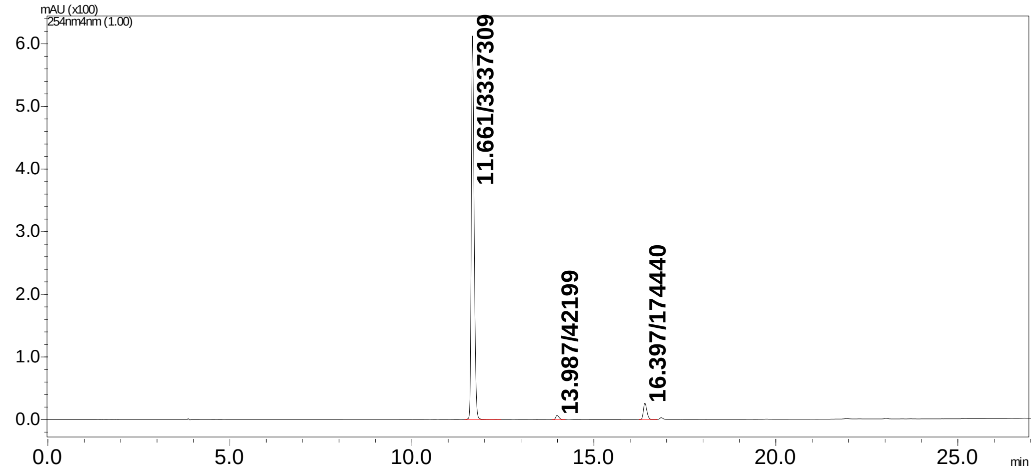

PILAB 3

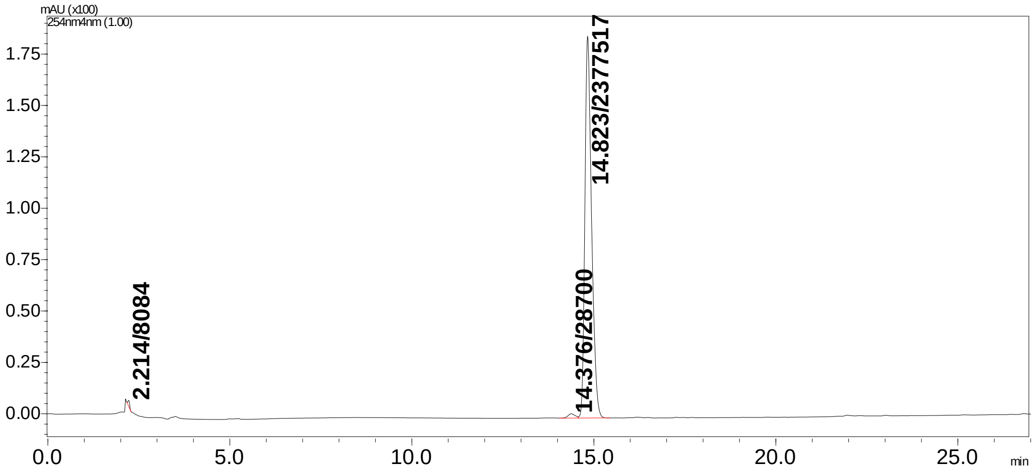

PILAB5

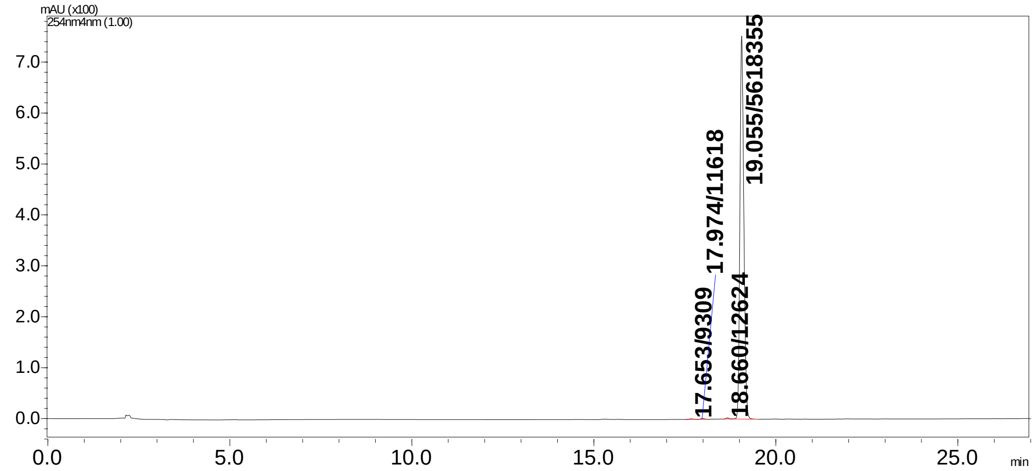

ZOOM

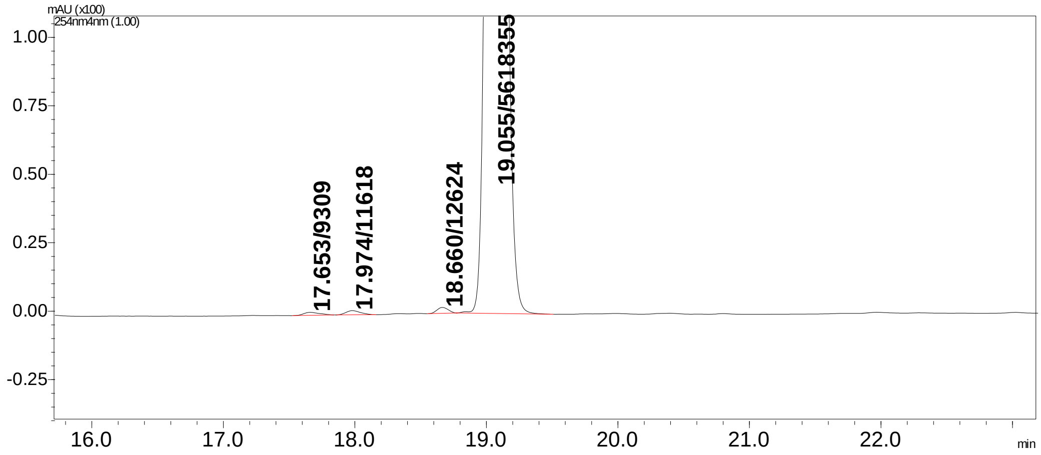

PILAB6

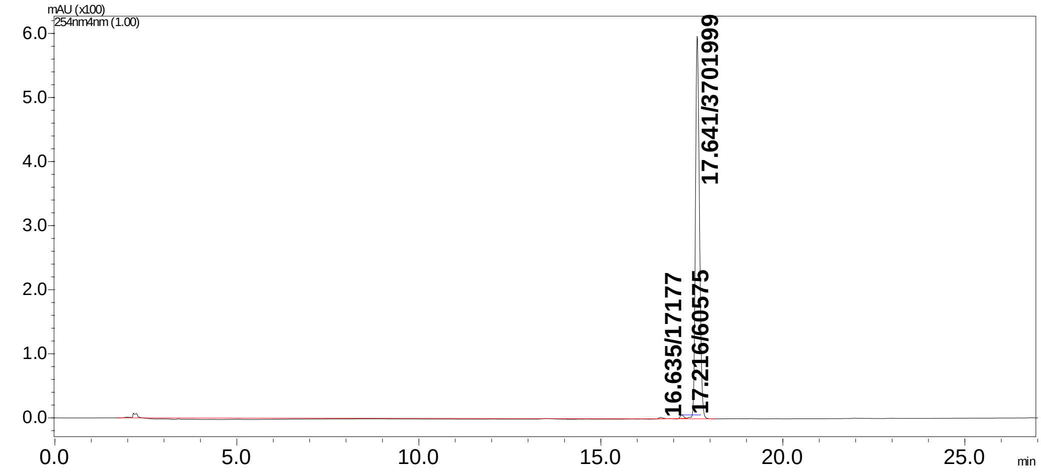

ZOOM

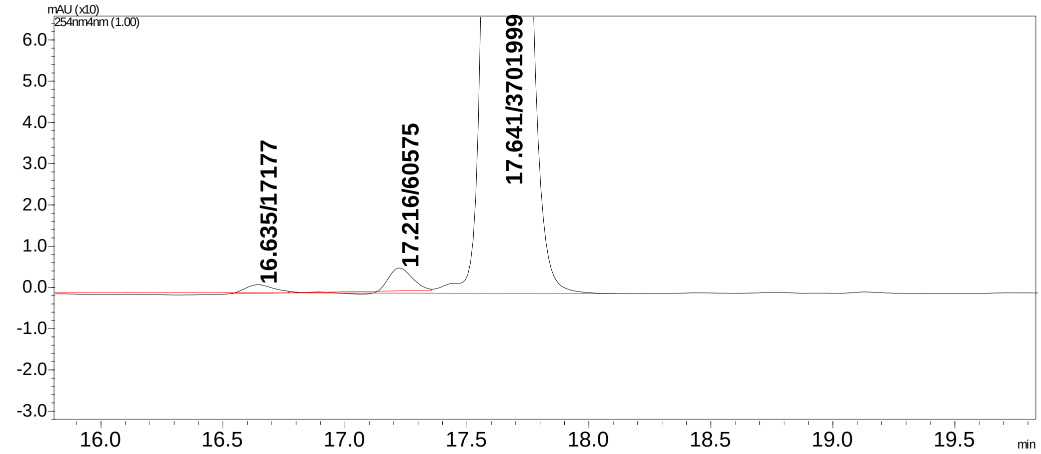

PILAB7

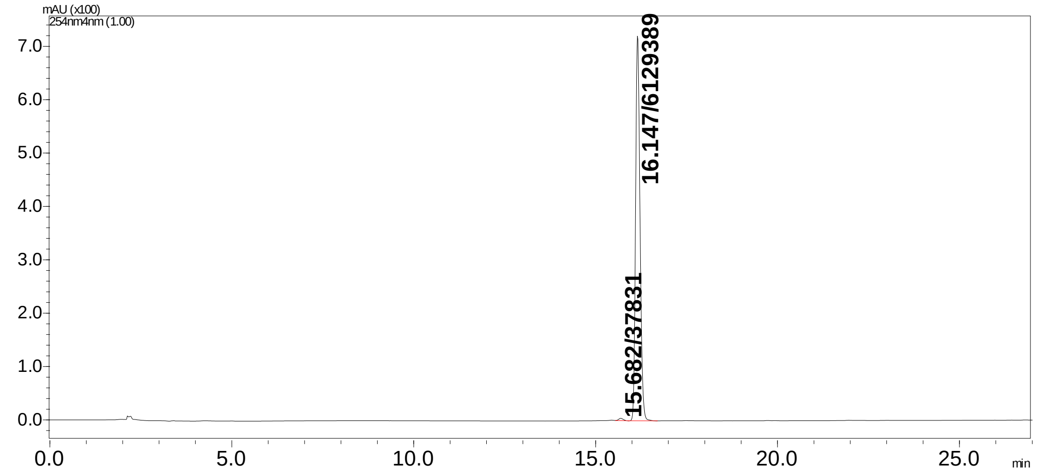

ZOOM

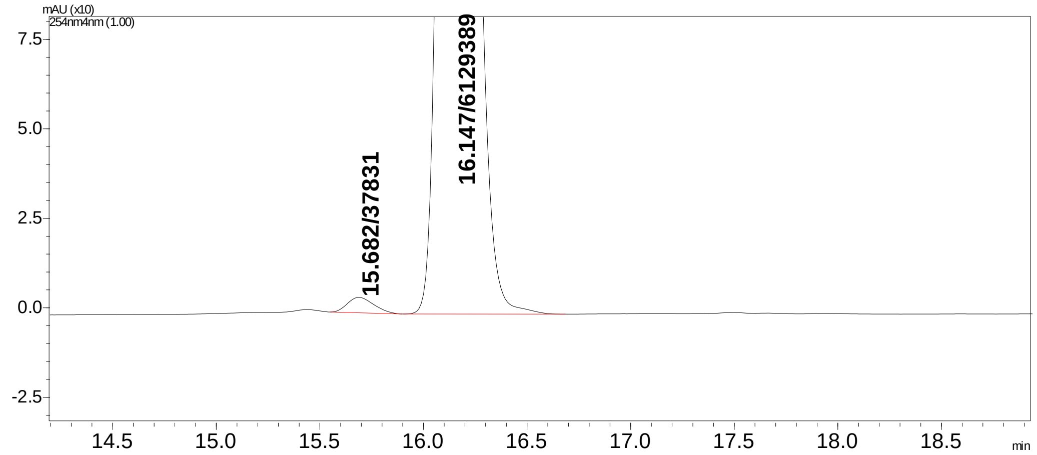

PILAB8

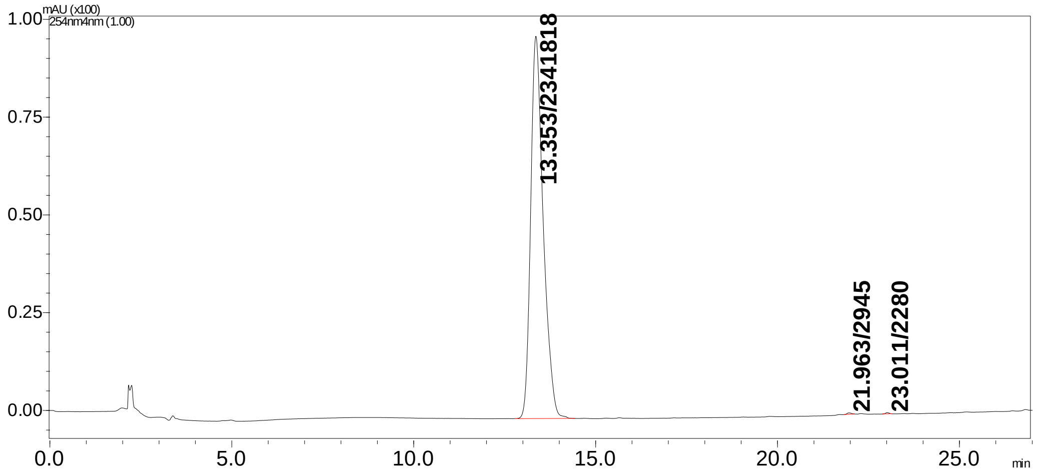

ZOOM

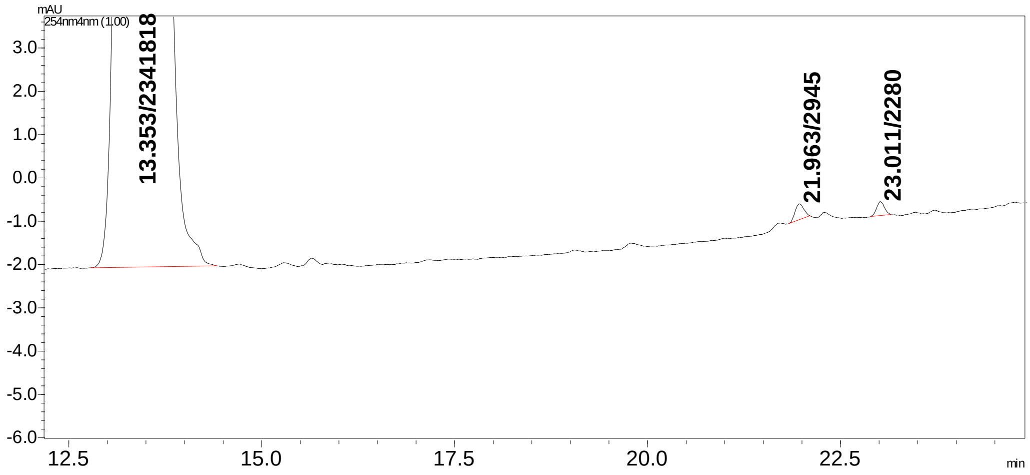

PILAB9

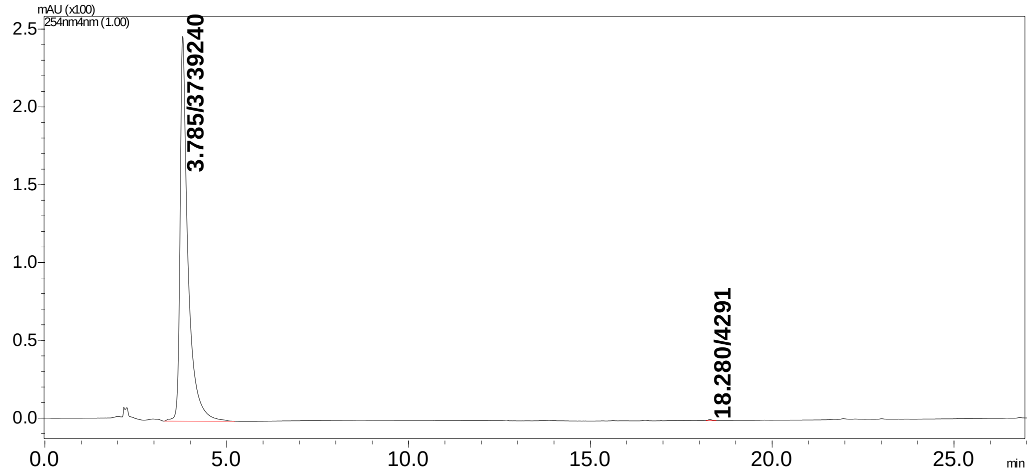

ZOOM

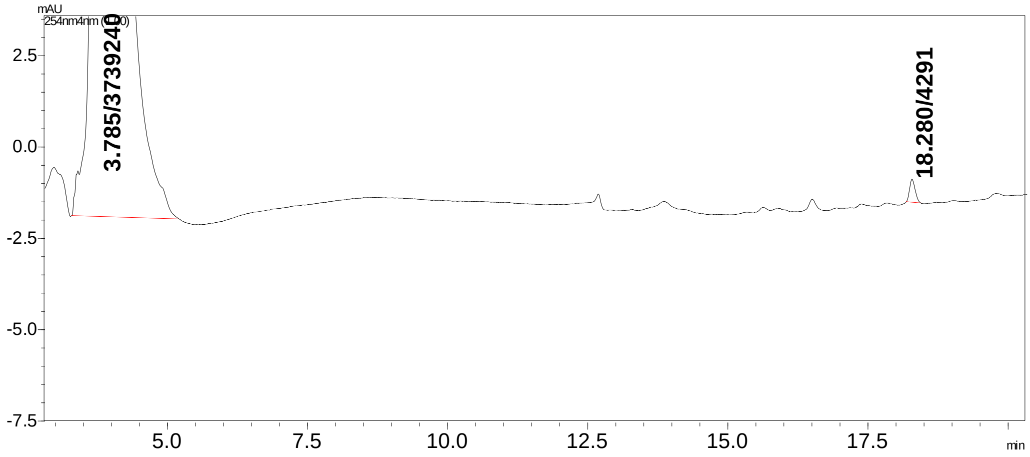

PILAB10

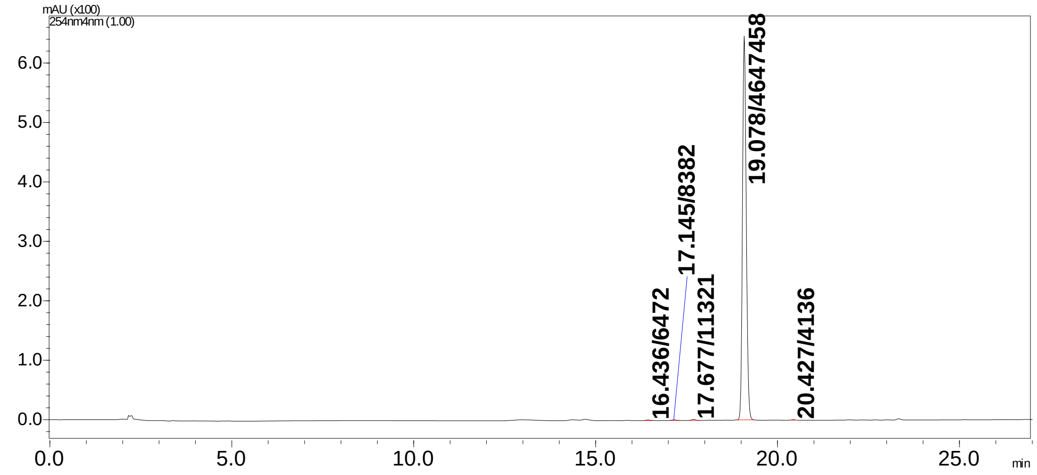

ZOOM

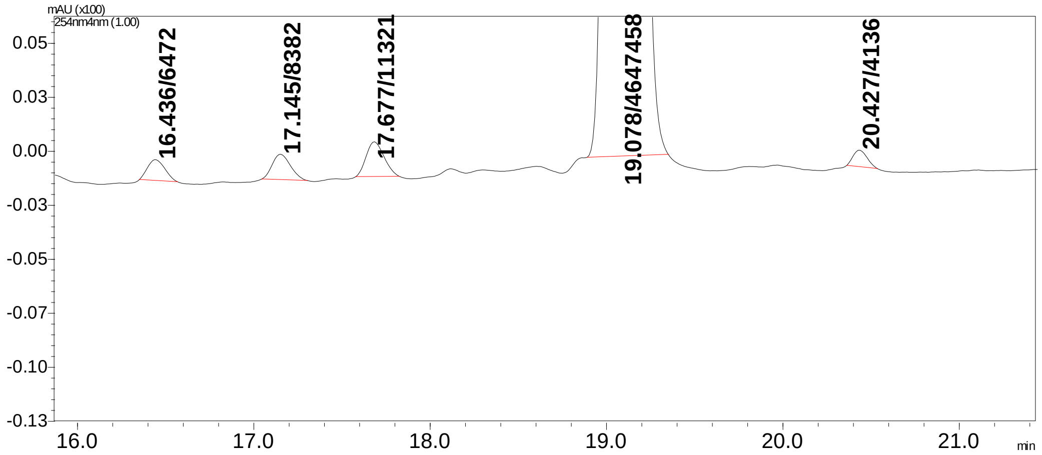

PILAB12

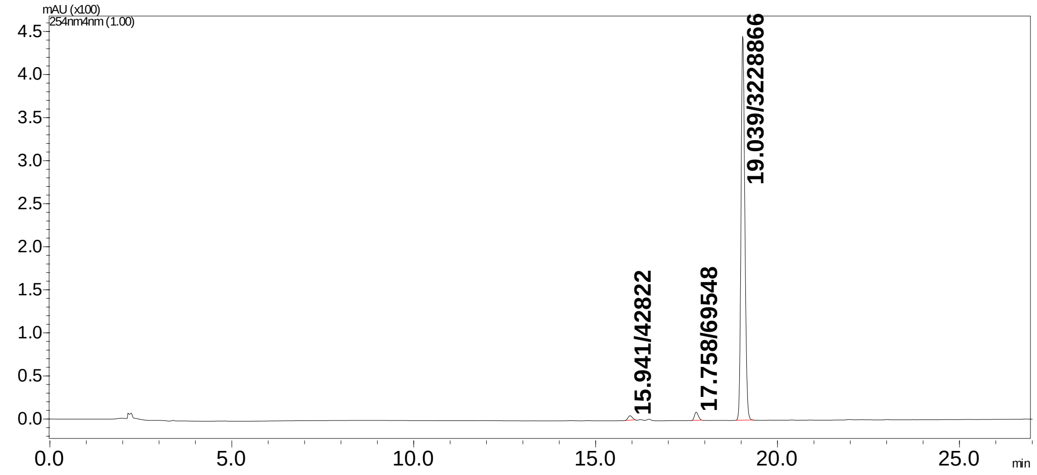

ZOOM

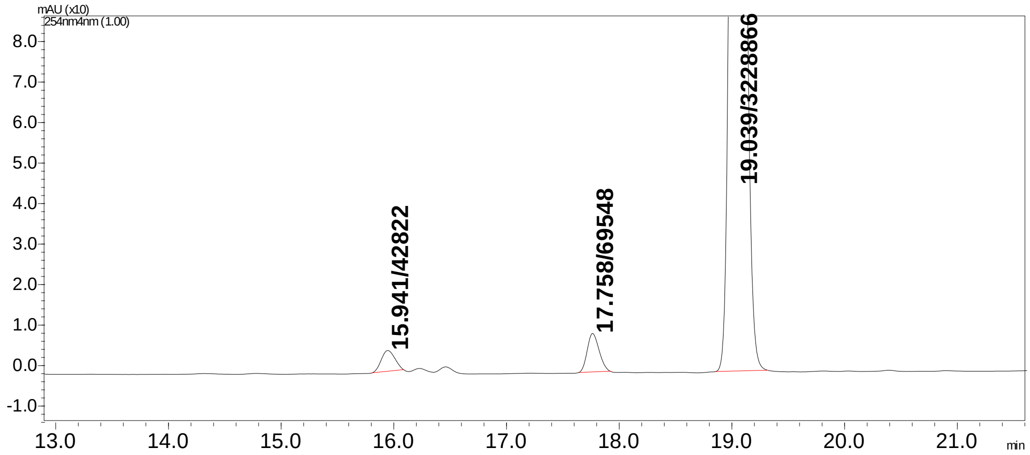

mAU (x10)
254nm4nm (1.00)
8.0
7.0
6.0
5.0
4.0
3.0
2.0
1.0
0.0
-1.0
15.941/42822
17.758/69548
19.039/3228866
13.0
14.0
15.0
16.0
17.0
18.0
19.0
20.0
21.0
min
